# Development, implementation and evaluation of an online course on evidence-based healthcare for consumers

**DOI:** 10.1186/s12913-020-05759-5

**Published:** 2020-10-08

**Authors:** Genie Han, Musa Mayer, Joseph Canner, Kristina Lindsley, Reva Datar, Jimmy Le, Annette Bar-Cohen, Janice Bowie, Kay Dickersin

**Affiliations:** 1 Department of Health, Behavior and Society, Johns Hopkins Bloomberg School of Public Health, 624 N Broadway, 707, Baltimore, MD 21205 USA; 2 Department of Epidemiology, Johns Hopkins Bloomberg School of Public Health, 615 N Wolfe St, E6012, Baltimore, MD 21205 USA; 3 Metastatic Breast Cancer Alliance, New York, USA; 4 Johns Hopkins Medicine, Baltimore, USA; 5 IBM Watson Health, New York, USA; 6 School of Public Health, University of Maryland College Park, College Park, USA; 7 National Breast Cancer Coalition, Washington, D.C, USA

**Keywords:** Patient education, Consumer education, Consumer health information, Distance education, Online learning

## Abstract

**Background:**

Evidence-based healthcare (EBHC) principles are essential knowledge for patient and consumer (“consumer”) engagement as research and research implementation stakeholders. The aim of this study was to assess whether participation in a free, self-paced online course affects confidence in explaining EBHC topics. The course comprises six modules and evaluations which together take about 6 h to complete.

**Methods:**

Consumers United for Evidence-based Healthcare (CUE) designed, tested and implemented a free, online course for consumers, *Understanding Evidence-based Healthcare: A Foundation for Action (“Understanding EBHC”).* The course is offered through the Johns Hopkins Bloomberg School of Public Health. Participants rated their confidence in explaining EBHC topics on a scale of 1 (lowest) to 5 (highest), using an online evaluation provided before accessing the course (“Before”) and after (“After”) completing all six course modules. We analyzed data from those who registered for the course from May 31, 2007 to December 31, 2018 (*n* = 15,606), and among those persons, the 11,522 who completed the “Before” evaluation and 4899 who completed the “After” evaluation. Our primary outcome was the overall mean of within-person change (“overall mean change”) in self-reported confidence levels on EBHC-related topics between “Before” and “After” evaluations among course completers. Our secondary outcomes were the mean within-person change for each of the 11 topics (mean change by topic).

**Results:**

From May 31, 2007 to December 31, 2018, 15,606 individuals registered for the course: 11,522 completed the “Before” evaluation, and 4899 of these completed the “After” evaluation (i.e., completed the course). The overall mean change in self-reported confidence levels (ranging from 1 to 5) from the “Before” to “After” evaluation was 1.27 (95% CI, 1.24–1.30). The mean change by topic ranged from 1.00 (95% CI, 0.96–1.03) to 1.90 (95% CI, 1.87–1.94).

**Conclusion:**

Those who seek to involve consumer stakeholders can offer *Understanding EBHC* as a step toward meaningful consumer engagement. Future research should focus on long-term impact assessment of online course such as ours to understand whether confidence is retained post-course and applied appropriately.

## Background

Patients and consumers (“consumers”) are increasingly valued as stakeholders in research and research implementation, such as when determining study design, performing grant review and developing systematic reviews and clinical practice guidelines [1–7]. Consumer engagement has been found to improve study enrollment and retention, likelihood of funding and inclusion of patient-centered outcomes; it may even help to reduce research waste [8–10]. More importantly, the inclusion of consumers as stakeholders is essential because they have priorities and perspectives that are often not reflected in current research and clinical practice guidelines, although consumers are the focus of many healthcare interventions [11–16].

To encourage research production relevant to end-users and their healthcare decision-making, the National Academy of Medicine (NAM) (formerly the Institute of Medicine) has published several reports that recommend consumer engagement [5, 6, 17]. Government agencies and international funders such as the National Health and Medical Research Council in Australia, Patient-Centered Outcomes Research Institute (PCORI) and the Agency for Healthcare Research and Quality (AHRQ) in the United States and the international Wellcome Trust encourage and sometimes require that funded projects include consumer stakeholder involvement [18–21].

One purported obstacle to consumer engagement is that consumers lack scientific background [4, 22]. This criticism may include lack of knowledge about evidence-based healthcare (EBHC), defined by the Joanna Briggs Institute as “decision-making that considers the feasibility, appropriateness, meaningfulness and effectiveness of healthcare practices” [23–26].

Training consumer stakeholders in evidence appraisal, research design and similar EBHC topics is recommended by PCORI, AHRQ, NAM, among others [5–7, 27, 28]. We believe that for meaningful consumer engagement to take place, training of consumer stakeholders in EBHC is required. Further, we believe that consumers engaged in the research process should be able to understand the language of EBHC and yet not become so immersed in science that they lose the consumer perspective.

Consumers United for Evidence-based Healthcare (CUE), a national consumer advocacy coalition in the United States, launched a free online course in 2007 to help consumers understand the fundamentals of EBHC. CUE does not accept any industry funding and is funded by AHRQ and PCORI. The objective of our study is to describe an online course on EBHC for consumers and to examine its reported impact on confidence in explaining the covered topics (“confidence”) among course completers in the 11-year period since its launch. Our analysis focused on course completers, as privacy concerns prevented us from contacting non-completers regarding reasons for attrition.

## Methods

### Development of an online course for consumers

Between 2005 and 2007, with funding from AHRQ, CUE developed a web-based distance learning course titled *Understanding Evidence-based Healthcare: A Foundation for Action (“Understanding EBHC”)*. CUE and the Johns Hopkins Bloomberg School of Public Health’s Center for Teaching & Learning (CTL) staff provided input to the course as it was being developed. The CTL maintains the course hosting platform (currently CoursePlus, www.courseplus.jhu.edu/core/index.cfm/go/course.home/cid/1739/, last accessed April 3, 2020) and collects user activity data (e.g., date of course registration, number of modules completed). We made the course available to the public in May 2007, with periodic updates to clarify language that did not affect the content of the course. In 2011, a supplemental course titled *The FDA and the Regulation of Healthcare Interventions* was added as an adjunct to the web course.

*Understanding EBHC* illustrates key topics with real-world examples. An experienced consumer advocate (MM) prepared the first and subsequent versions of the course for consumers with feedback at each stage from KD. The course comprises six audiovisual lecture modules:

1. *Introduction*. What is evidence-based healthcare and why is it important?
2. *Ask.* The importance of research questions in evidence-based healthcare;
3. *Align.* Research design, bias and levels of evidence;
4. *Acquire*. Searching for healthcare information; assessing harms and benefits
5. *Appraise.* Behind the numbers: Understanding healthcare statistics; Science, speed and the search for best evidence; and
6. *Apply.* Critical appraisal: Making better decisions for evidence-based healthcare, Determining causality.
  A detailed course outline is provided in Additional file 1.

### Registration and “before” and “after” evaluations

When a participant registers for the course through the online hosting platform, the CTL assigns them a unique User ID and provides a mandatory participant information survey that asks for name, email address and country of residence. Participants can access course content once they complete a required “Before you begin” (“Before”) evaluation developed by CUE and JHSPH staff. We use the term “evaluation” to refer to forms developed by CUE and JHSPH staff, although the CTL uses the term “survey” to refer to both their participant information survey and our evaluations. The “Before” evaluation includes questions on demographics (e.g., sex and race/ethnicity), level of involvement in health advocacy and reason for taking the course. “Before” evaluation participants are also asked to rate their confidence in explaining to the 11 EBHC topics covered in the course to a friend, using Likert scale ratings from 1 (1 = lowest) to 5 (5 = highest). The 11 topics are:

1. Systematic review;
2. Evidence-based healthcare;
3. Cochrane Collaboration;
4. How to find research articles using PubMed (MEDLINE);
5. How to use online sources (e.g., The Cochrane Library) to find summaries of existing research evidence;
6. Reasons why high quality systematic reviews are more useful than individual studies for understanding whether a treatment works;
7. How researchers assess whether a research study’s results might be due to chance;
8. How to assess whether a research study’s results might be explained by bias;
9. Why randomizing patients in a clinical trial makes us more confident that the groups being compared are similar;
10. How to assess whether an exposure might be causing an outcome or whether it might be associated with the outcome; and
11. Why it’s important that scientists publish results from ALL, not just some, of their research.

After participants complete all six course modules, they take the “After you complete” (“After”) evaluation, which again asks for a rating of confidence. Completion of the “After” evaluation is necessary to receive a course certificate. To assess confidence after completing the course, we analyzed only those with both “Before” and “After” evaluations.

The course and evaluations together take about 6 h to complete and can be done in 10- to 15-min segments. Evaluation forms are available in Additional files 2 and 3.

### Consumer involvement while developing the course

At several junctures in the development process we presented learning modules to the public for feedback, including at conferences such as the annual Cochrane Colloquia [29–31]. Feedback included written evaluations and in-person discussion between consumers and course developers. We revised the course in response to feedback, such as requests for more graphics in the slide material. We also regularly made drafts of the modules available to CUE member organizations and requested their input.

### Analysis of participant data

**Fig. 1:**
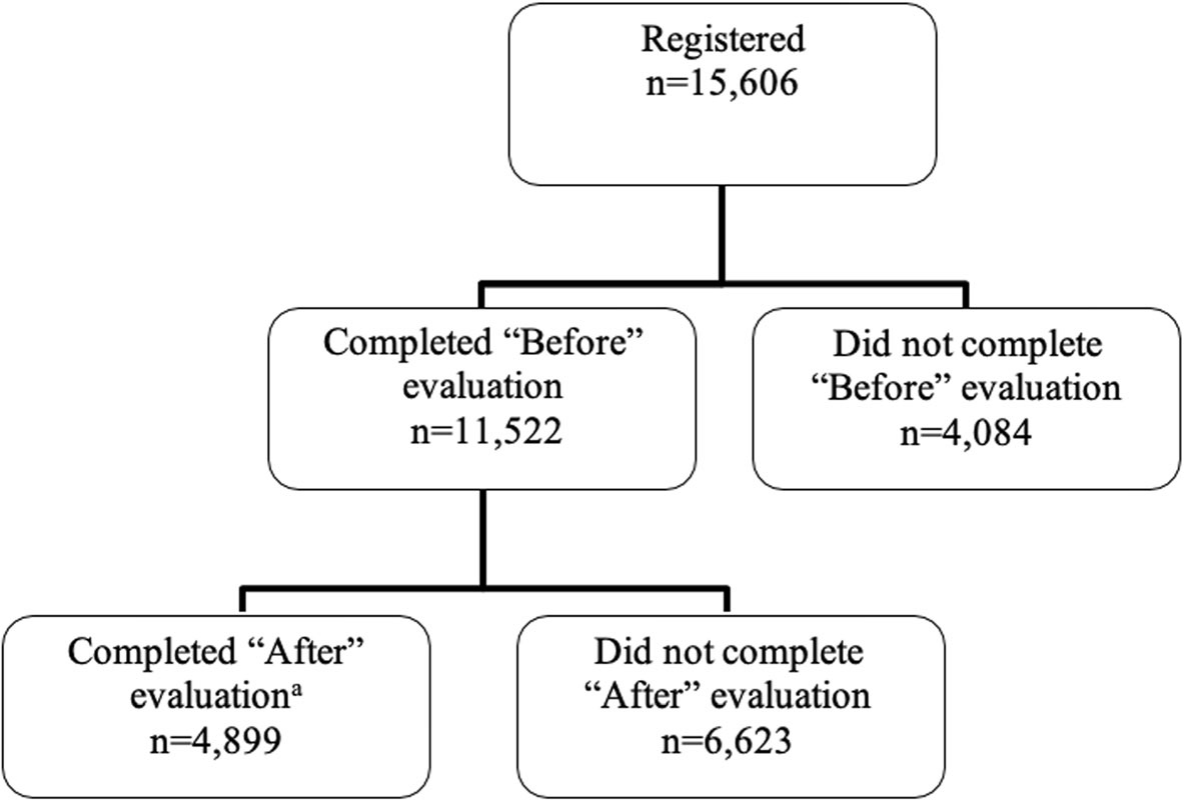
Numbers of participants in *Understanding EBHC* between May 31, 2007 and December 31, 2018. ^a^Termed “course completers”

We analyzed participant data for those who registered for the course between May 31, 2007 to December 31, 2018. This paper presents data obtained from three sources: the participant information survey and the “Before” and “After” evaluations. We completed Fig. 1 using participant activity data; Table 1 using participant information survey responses; and Tables 2 and 3 using “Before” and “After” evaluation responses. We matched “Before” and “After” evaluation responses using assigned User IDs to assess within-person changes in confidence levels.

**Table 1:** Participant characteristics, from “Before you begin” evaluation

	Completed “Before” evaluation		Completed “After” evaluation^**a**^		Did not complete “After” evaluation^**b**^	
	No.	(%)^c^	No.	(%)	No.	(%)
Total respondents	11,522	(100.0)	4899	(100.0)	6623	(100.0)
Age^d^						
Under 20	150	(1.3)	36	(0.7)	114	(1.7)
20–29	3869	(33.6)	1788	(36.5)	2081	(31.4)
30–39	3059	(26.6)	1308	(26.7)	1751	(26.4)
40–49	2163	(18.8)	927	(18.9)	1236	(18.7)
50–59	1583	(13.7)	601	(12.3)	982	(14.8)
≥ 60	571	(5.0)	190	(3.9)	381	(5.8)
Prefer not to answer or no response	127	(1.1)	49	(1.0)	78	(1.2)
Sex/gender						
Female	8672	(75.3)	3927	(80.2)	4745	(71.6)
Male	2563	(22.2)	870	(17.8)	1693	(25.6)
Prefer not to answer or no response	287	(2.5)	102	(2.1)	185	(2.8)
Race/ethnic origin^e^						
African American/Black	1007	(8.7)	454	(9.3)	553	(8.3)
American Indian or Alaskan Native	35	(0.3)	14	(0.3)	21	(0.3)
Asian or Pacific Islander	805	(7.0)	261	(5.3)	544	(8.2)
Caucasian/White	6706	(58.2)	2806	(57.3)	3900	(58.9)
Indian/Pakistani	477	(4.1)	116	(2.4)	361	(5.5)
Latino, Latina/Hispanic	1361	(11.8)	891	(18.2)	470	(7.1)
Middle Easterner	366	(3.2)	78	(1.6)	288	(4.3)
Other	196	(1.7)	60	(1.2)	136	(2.1)
Prefer not to answer or no response	569	(4.9)	219	(4.5)	350	(5.3)
Highest level of education completed						
Some high school, high school diploma, or equivalent	350	(3.0)	119	(2.4)	231	(3.5)
Some college (no degree)	982	(8.5)	473	(9.7)	509	(7.7)
Trade/technical school	77	(0.7)	27	(0.6)	50	(0.8)
Associate degree	1209	(10.5)	778	(15.9)	431	(6.5)
Bachelor’s degree or higher (eg, doctorate)	8687	(75.4)	3434	(70.1)	5253	(79.3)
Prefer not to answer or no response	217	(1.9)	68	(1.4)	149	(2.2)
Employment status						
Employed for an income	7671	(66.6)	3366	(68.7)	4305	(65.0)
Working as a volunteer	160	(1.4)	56	(1.1)	104	(1.6)
Not employed or not working	2039	(17.7)	833	(17.0)	1206	(18.2)
Other	1214	(10.5)	490	(10.0)	724	(10.9)
Prefer not to answer or no response	438	(3.8)	154	(3.1)	284	(4.3)
Reason for taking the course						
Training	846	(7.3)	272	(5.6)	574	(8.7)
Education	6412	(55.7)	3782	(77.2)	2630	(39.7)
Personal growth	3451	(30.0)	584	(11.9)	2867	(43.3)
Other or none of the above	679	(5.9)	197	(0.4)	482	(7.3)
Not reported	134	(1.2)	64	(1.3)	70	(1.1)

^a^ Completed both “Before” and “After” evaluations (“course completers”)

^b^ Completed “Before” evaluation, but not “After” evaluation (“course non-completers”)

^c^ Columns may not total 100% because of rounding

^d^ All differences in participant characteristics between course completers and course non-completers were found to be significant (*p* < 0.001)

^e^ Race and ethnic origin were grouped together in a single question

**Table 2:**
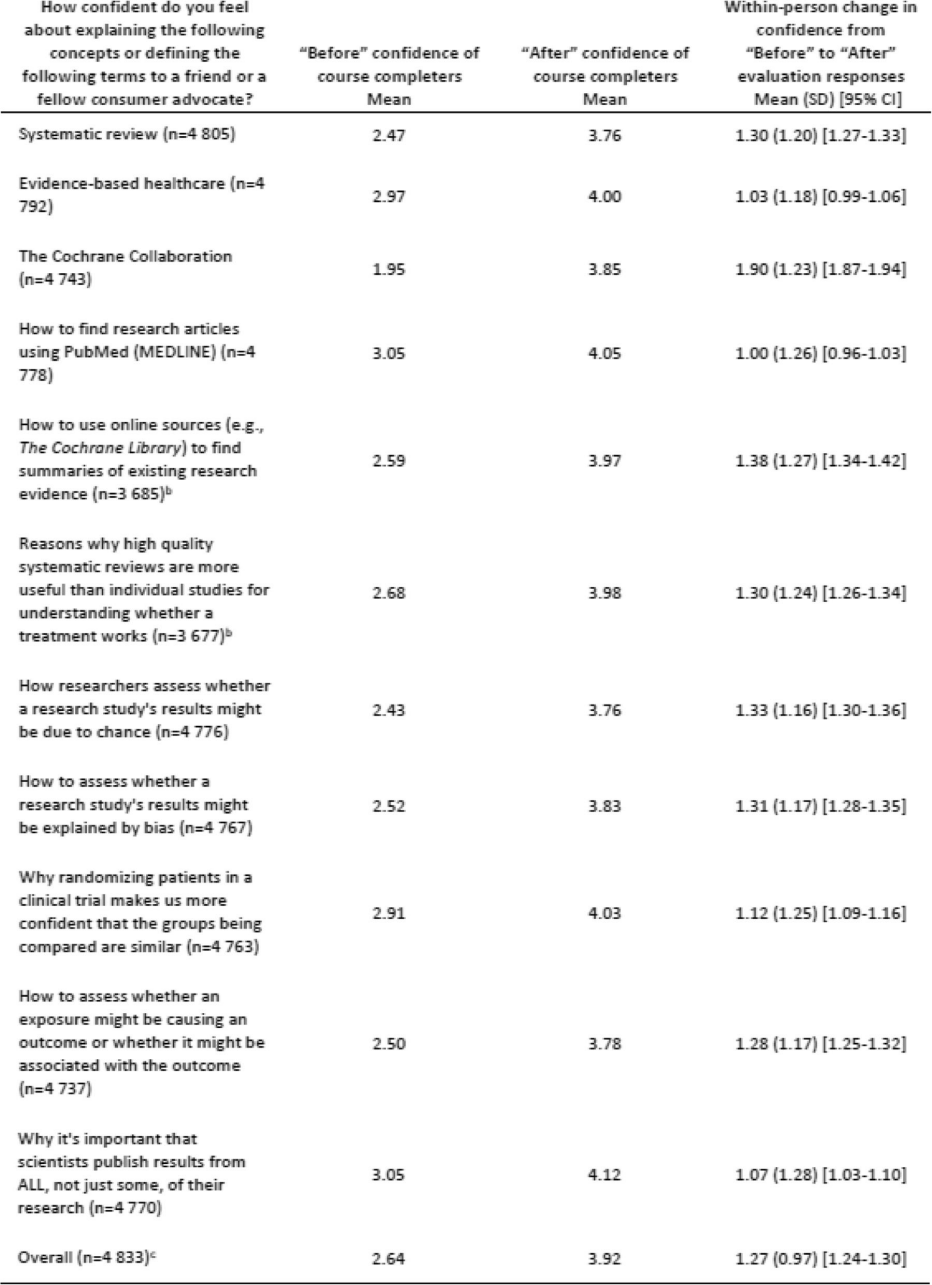
Mean confidence levelsa and mean within-person change observed by topic on “Before” and “After” evaluations

**Table 3:** Confidence levels on EBHC from “Before” to “After” evaluations

In terms of your knowledge on evidence-based healthcare, what is your confidence?^**a,b**^	“After” confidence of course completers						Total^**c**^	
	Not so confident		Moderately confident		Very confident			
**“Before” confidence of course completers**	No.	(%)	No.	(%)	No.	(%)	No.	(%)
Not so confident	81	(5.7)	1134	(80.5)	194	(13.8)	1409	(100)
Moderately confident	42	(1.4)	2051	(67.2)	961	(31.5)	3054	(100)
Very confident	2	(0.6)	125	(35.1)	229	(64.3)	356	(100)
**Total**	125	(2.6)	3310	(68.7)	1384	(28.7)	4819^d^	(100)

^a^ Data presented as paired observations

^b^ This question did not offer a “Prefer not to respond” answer choice

^c^ Changes from “Before” to “After” were found to be significant (*p* < 0.0001)

^d^ Although the online hosting platform prompted participants to answer all evaluation questions before proceeding, we encountered 80 missing responses

Our primary outcome was the overall mean of within-person change (“overall mean change”) in self-reported confidence levels on EBHC-related topics between “Before” and “After” evaluations among course completers. We obtained overall mean change by averaging across individuals the difference between the “Before” and “After” confidence level ratings for each of the 11 topics for each participant. Although we had “Before” and “After” evaluation data on a participant’s overall confidence about his or her knowledge of EBHC, these data were categorical (“not so confident,” “moderately confident,” and “very confident”) and could not easily be compared with numerical data. For this reason, we report change from “Before” to “After” in Table 3 and not as the primary outcome.

Our secondary outcomes were the mean within-person change for each of the 11 topics (“mean change by topic”). For both primary and secondary outcomes, we used a paired t-test to analyze the within-person change in self-reported confidence levels from the “Before” and “After” evaluations. We also reported standard deviation and 95% confidence interval for each outcome. Descriptive statistics of participant characteristics were obtained and reported as numbers and percentages. We compared differences in participant characteristics using Pearson’s χ^2^ test (Table 1). We conducted Bowker’s test for symmetry to determine whether a participant’s overall confidence about his or her knowledge of EBHC changed from “Before” to “After” (Table 3) [32]. All statistical analyses were performed using Stata/MP version 14.2 (Stata Corp, College Station, Texas).

In calculating the number of course participants and conducting analyses, we included each participant only once. If a participant took the course more than once (“course re-taker”), we only analyzed data from her or his first attempt. We identified course re-takers using unique User IDs assigned by the course hosting platform and the participant’s email address. In the case that an individual re-registered using a different email address and obtained a different User ID, two authors (GH, JC) independently classified participants as likely re-takers using reported name, gender and race/ethnicity. Differences in classification were resolved through discussion.

## Results

Figure 1 presents a participant flow diagram, starting at registration (*n* = 15,606), then taking the “Before” evaluation (*n* = 11,522 [73.8%]) and lastly taking the “After” (*n* = 4899 [31.4%]) evaluation.

### Characteristics of “before” evaluation completers (Table 1)

Most of those completing the “Before” evaluation were under 40 years of age (61.5%), female (75.3%) and had a bachelor’s degree or higher (75.7%).

### Characteristics of course completers versus that of non-completers (Table 1)

Course completers had a different set of characteristics compared to non-completers. Notable differences were as follows: a higher percentage of course completers identified as Latino, Latina/Hispanic (completers vs. non-completers, 18.2% vs. 7.1%); had an Associate degree (15.9% vs. 6.5%); and lived in North America (91.7% vs. 56.2%) (Additional file 4).

### Confidence levels in EBHC topics (Table 2)

The overall mean change in self-reported confidence levels from the “Before” to “After” evaluation was 1.27 (95% CI, 1.24–1.30). The mean change by topic for the 11 topics ranged from 1.00 to 1.90. All mean changes were statistically significant (*p* < 0.001).

“How to find research articles using PubMed (MEDLINE)” had the lowest mean change (mean, 1.00; 95% CI, 0.96–1.03) and the highest mean “Before” confidence level (3.05). “What is the Cochrane Collaboration?” had the highest mean change (mean, 1.90; 95% CI, 1.87–1.94).

### Confidence levels in EBHC (Table 3)

Most course completers felt moderately confident in their knowledge of EBHC in the “Before” (3054/4819 [63.4%]) and “After” (3310/4819 [68.7%]) evaluations. In the “Before” evaluation, there were 356/4819 (7.4%) course completers who reported “Very confident” in their knowledge of EBHC, which improved to 1384/4819 (28.7) in the “After” evaluation. Conversely, 1409/4819 (29.2%) course completers reported “Not so confident” in the “Before” evaluation, compared to 125/4819 (2.6%) in the “After” evaluation.

## Discussion

Our results suggest that *Understanding EBHC* is an effective educational offering for consumers who wish to increase their confidence in explaining the basic topics of EBHC. Using repeated measurements for data collection and analysis for 4899 course completers, we found that the online training *Understanding EBHC* course helped participants increase their overall confidence on EBHC topics. We were gratified that this finding was also found in each of the 11 individually assessed topics; the course had highest within-person change explaining Cochrane and how to use the *Cochrane Library*. After completing the course, participants reported the least confidence in explaining systematic reviews and how study results may be due to chance. Participants reported the most confidence in explaining the importance of publishing all research results and how to search PubMed.

We were able to still collect several interesting observations on non-completers using participant activity data. Out of 6623 non-completers, over one-third did not complete any module (*n* = 2421) and over half completed between one to five modules (*n* = 3717). Moreover, the number of participants completing each module decreased steadily from Modules 1 to 6, suggesting that modules were taken in chronological order or increased in difficulty. In regard to the latter, we received feedback during development that Modules 5 and 6 were particularly challenging as they contained statistical material.

We also encountered total (i.e., no questions were answered) or partial (i.e., some questions were answered) missing data on the “Before” and “After” evaluations, although all survey and evaluation questions were required. For example, there were 66/4899 (1.3%) course completers who did not answer any questions in the “After” evaluation. Of the course completers who answered at least one question in the “After” evaluation, response rates on individually assessed topics varied between 76.1% (3677/4833) to 99.4% (4803/4833). Although we can only speculate on why total or partial missing data occurred, we do not expect that that the missing data will affect our conclusions.

We believe that our findings are important for researchers because training consumer stakeholders in preparation for research engagement has been demonstrated to provide benefit for both parties [27, 28, 33]. Specifically, it has been found that researchers obtain more meaningful consumer input [28, 33], whereas consumers have increased knowledge about research and feel more comfortable with contributing their perspective [27, 34]. When training is not offered, or is not offered in easy to understand format, researchers risk overburdening consumers who may feel too inexperienced to engage in discussions with other stakeholders [22].

Despite the demonstrated benefit of training consumer stakeholders (e.g., on improving consumer engagement), researchers may still be wary about spending resources on training [8, 22, 27, 35]. Mullins et al. hypothesize that researchers will develop more efficient methods of consumer engagement, which will decrease the associated time and cost [35]. *Understanding EBHC* and similar public trainings offered free of charge where possible, online and in-person, can support this need. Future trainings could compare course development with and without consumer stakeholders to better assess the impact of their involvement.

Online and in-person trainings with similar goals and target audiences to that of *Understanding EBHC* have observed positive short-term impact consistent with our findings. For example, a randomized controlled trial found that participants with access to web portal resources had more positive attitudes about EBHC-related skills, such as searching for health information, compared to those with no resources [36]. An in-person training offered by the National Breast Cancer Coalition also observed increased confidence in explaining EBHC topics immediately following their 5-day in-person course [37]. The disadvantage to in-person trainings is that they require time and money (e.g., lodging), which is already in short supply for consumers [8, 22]. Online trainings are appealing for maintaining a level of interactivity while allowing for flexibility in when and where participants take them. Additional funding may explore blended learning (a combination of online and in-person training), which has been shown to be more effective in improving student learning outcomes than online only or in-person only learning [38].

Although we found an increase in participant EBHC-related topic confidence following our online course, we are uncertain as to the long-term impact of educational resources on EBHC for consumers [8, 34, 39]. For example, we could not contact course completers to ask them additional questions and assess how long course participants maintain confidence in EBHC principles. Berger et al. sought to evaluate the long-term impact of an in-person course on EBHC and discovered that the level of resulting implementation varied greatly among participants [40]. In addition, our confidence assessments rely on self-reported evaluation data. We did not define a mean change value in self-reported confidence levels that would constitute improved understanding. Future work could involve supplementing self-reported data with objective measures of knowledge, such as quizzes [36, 40].

Importantly, course completion data suggest that *Understanding EBHC* course material is appropriate for and appeals to diverse introductory-level audiences. Although participants had different baseline levels of experience with EBHC, completion rates were not significantly different between those with experience and those without. We infer that *Understanding EBHC* is a promising educational intervention for individuals regardless of prior experience with EBHC.

Updates to the course will be necessary in the near future, to integrate recent examples, introduce objective measures of knowledge and potentially translate course offerings into other languages. Updaters should consider which content areas can be improved, for example, topics where the “After” evaluation indicates lower confidence.

Ensuring course accessibility and relevance among priority populations (such as racial and ethnic minorities, individuals with low-income, rural residents) is paramount and requires dedicated teaching and administrative staff. The most pressing challenges as we move forward with this course are finding resources to keep *Understanding EBHC* up-to-date and disseminating information about the course so that those who would benefit from its offerings are aware of its availability. From our study, we know that *Understanding EBHC* reaches a variety of individuals, racially, ethnically, geographically and educationally. We also know, however, there is an opportunity to improve course completion rates for individuals who identify as part of a priority population by developing more expansive dissemination methods and course updates.

## Conclusion

*Understanding Evidence-based Healthcare (EBHC): A Foundation for Action* is a free online training on EBHC specifically geared toward consumers. Our findings indicate that completing the course increased participants’ confidence on EBHC topics. Researchers who seek to contribute to the partnership with and engagement of consumers may do so by recommending *Understanding EBHC*. Future research should be directed toward assessing long-term course impact on consumer contributions and engagement with respect to EBHC.

## Supplementary information

**Additional file 1. **Concepts and examples covered in *Understanding Evidence-based Healthcare.* Course module-specific learning objectives and examples.

**Additional file 2.** Survey: Participant Information – before you begin course. Survey form provided to course participants prior to accessing the course.

**Additional file 3.** Survey: Participant Information – after you complete course. Survey form provided to course participants after completing the course.

**Additional file 4.** Characteristics of “Before you begin” survey completers (an expansion of published Table 1). All collected data on participant characteristics.

## Data Availability

The data that support the findings of this study are available on request from the corresponding author GH. The data are not publicly available due to them containing information that could compromise research participant privacy/consent.

## Abbreviations

EBHC: Evidence-based healthcare
