# Supplementary material for: Development, implementation and evaluation of an online course on evidence-based healthcare for consumers
Source: BMC Health Serv Res. 2020 Oct 8;20:928. doi: 10.1186/s12913-020-05759-5 (PMC7542874; doi:10.1186/s12913-020-05759-5)
Supplement: Supplementary file 1 — Additional file 1. Concepts and examples covered in Understanding Evidence-based Healthcare. Course module-specific learning objectives and examples. [file 12913_2020_5759_MOESM1_ESM.pdf]

Module	Key Concepts	Real-World Examples
Module 1: Introduction. What is Evidence Based Health Care and Why is it Important?<br><br>*A. What is Evidence-based Healthcare?*<br><br>*B. Seeking the Best Healthcare Evidence*	▪ **Key concepts: Research, data, evidence, randomized trials, systematic reviews, meta-analysis**<br>▪ Introduction to Kay Dickersin and Musa Mayer<br>▪ The need for evidence-based health care for making good health care decisions, and its importance to advocates<br>▪ Managing the ever-increasing amount of health care information means learning new skills for advocates<br>▪ Definition Evidence-based health care **(EBHC)**<br>▪ EBHC enhances clinical judgment and consumer choice, it doesn't replace it.<br>▪ Definitions: **research, data, evidence**<br>▪ What the public believes vs reality. Appreciation of the impact of **information overload**, the challenges in **staying up to date** with new research, limitations on health care providers' time.<br>▪ Randomized trials and the need for **systematic reviews**<br>▪ Brief introduction to **Cochrane Collaboration. Forest plots** explained.<br>▪ Introduction to the **learning curve** and where the course will be heading	***Cautionary tales:***<br>What can happen when information overload and other factors lead to existing research not being systematically reviewed and the results disseminated to health care providers<br><br>▪ ***JAMA* review of reviews on thrombolytic (clot-busting) drugs for myocardial infarction**<br><br>▪ **Cochrane logo explained: antenatal steroids in premature birth**
Module 2: ASK. The Importance of Research Questions in Evidence-based Healthcare<br><br>*A. Classifying Healthcare Questions*<br><br>*B. How to Formulate a Research Question*<br><br>*C. Why Asking the Right Research Question Matters*<br><br>*D. What led to the VIOXX Disaster and What Can We Learn From It?*	▪ **Key concepts: Why research questions matter, define and classify healthcare questions, demonstrate that the kind of research study depends on the kind of question, formulate healthcare questions**<br>▪ Questions as the heart of health care research; different types of questions require different types of research designs to provide the best evidence.<br>▪ We classify questions into nine categories: prevention, screening, diagnostic accuracy, prognosis, incidence, prevalence, etiology, therapy and harm.<br>▪ Learning to classify and formulate an effective research question is a critical component of getting the answer<br>▪ How to formulate questions using the **PICO** model: Population, Intervention, Comparison, Outcome. Example: **TEAS (transcutaneous electrical acupoint stimulation) for prevention of post-tonsillectomy nausea and vomiting in children**.<br>▪ In the context of the COX-2 cautionary tale:<br>- **Absolute risk, absolute risk difference, relative risk**<br>- Meta-analysis example of COX-2's for harm<br>- Early approval led to uptake before harm understood	***Cautionary tale:***<br>- What can happen when important questions or safety and efficacy are not asked or answered prior to broad marketing and use of a therapy.<br>- Well-designed studies ask the right questions about the safety and efficacy of health care interventions<br>- Failure to ask and answer appropriate research questions has a human cost.<br>- Impact of industry marketing to doctors and patients, as well as media hype.<br><br>▪ **Vioxx, the COX-2 inhibitors and NSAIDS**
Module 3: ALIGN. Research Design, Bias, and Levels of Evidence	▪ **Key concepts: Aligning the best study design to the clinical question: Experimental and observational research designs and concepts, problems and solutions in research design, levels of evidence, types**	***Cautionary tale:***<br><br>What can happen when there is little or

Module	Key Concepts	Real-World Examples
A. Randomized Controlled Trials (RCTs): Population, Intervention, Comparison, Outcome<br><br>*B. RCTS: Randomization*<br><br>*C. Placebos, Masking, and Bias*<br><br>*D. Levels of Evidence*<br><br>*E. Observational Studies*<br><br>*F. HRT: The Nurses' Health Study vs The Women's Health Initiative*<br><br>*G. The Women's Health Initiative*	**of observational studies**<br>▪ **RCTs**:<br>PICO:<br>Population: Every study is a **sample** of the population of interest<br>Intervention: differentiating **experimental** and **observational study design**<br>Comparison: importance of **controls**<br>Outcome: issues with **interim outcomes**<br>Design of RCTs (graphic)<br>Exploring issues in randomized controlled trials: **equipoise, randomization, placebos, masking (blinding), and bias (selection, performance, and information)**<br>▪ **Levels of evidence** for intervention questions: from unsystematic clinical observations, physiologic studies, observational studies, RCTs, to systematic reviews of RCTs<br>▪ Advocacy concerns with experimental studies that offer high levels of evidence—time to results, equal access, loss of control, etc.<br>▪ Observational study designs and examples: **ecologic studies, case report, case series, cross-sectional studies (surveys, prevalence studies), cohort and case control studies**<br>▪ Lower levels of evidence can be misunderstood and patients and public affected	no understanding of levels of evidence, allowing treatments to be based solely on findings from observational studies, rather than randomized, controlled trials. Commercial influence.<br><br>**Post-menopausal hormone therapy for the prevention of cardiovascular disease**<br><br>- The Nurses' Health Study<br>- A short history of menopausal hormone therapy<br>- The Heart and Estrogen/progestin Replacement Study (HERS) Study<br>- McPherson/Hemminki systematic review<br>- Women's Health Initiative<br>- Translating study results to women's lives
Module 4: ACQUIRE. Searching for Health Care Information. Assessing Harms and Benefits<br><br>*A. Searching the Internet for Health Care Information*<br><br>*B. Reporting Bias and a Practice Scenario for Searching*<br><br>*C. Using The Cochrane Library and Other Web Resources*<br><br>*D. Assessing Harms and*	**Key concepts: Evidence and where to look for it, search techniques and limitations, interpreting the information we find, forms of publication bias, risk and harm, assessing harms *and* benefits**<br>▪ Definitions: **Search, search strategy, publication bias, peer reviewed journal**.<br>▪ Credible sources of information. What is a good search and why does it matter?<br>▪ Learning where to look for evidence and effective search techniques as an essential skill for health care advocates<br>▪ Selection of **search terms**, search **limits**<br>▪ The basics of conducting searches and the limitations of doing your own searching<br>▪ **Reporting bias**, or how do we know we've found everything that's relevant?<br>▪ Practice searches, using brief scenarios<br>▪ Issues of full text and **open access**<br>▪ How to seek out primary sources and high-quality secondary sources and	***Cautionary tales:***<br><br>What can happen when researchers are unable to find critical information from in health care research.<br>▪ **Hexamethonium**<br>Demonstrate a search example of bedwetting<br>▪ **Bedwetting**<br>List and assess Internet sites for searching<br>▪ **PubMed, Google, ClinicalTrials.gov, TrialsCentral.org, *The Cochrane Library***

**Module**	**Key Concepts**	**Real-World Examples**
Benefits<br><br>*E. Assessing Harms and Benefits of Cancer Screening*	factors to consider<br>▪ **Definitions: risk, harm, benefit, screening**<br>▪ Learning to question both the harms and benefits of cancer screening<br>▪ Definitions: **Sensitivity, specificity**. How changing a cut-off value for a positive screening test can results in more cases identified but also more false positives<br>▪ Definitions: **Incidence, prevalence**<br>▪ Overestimation of risk for disease and benefits of treatment in relation to marketing and media coverage	What can happen when broad use of biomarker tests is used for screening for cancer<br>▪ **PSA and Prostate Cancer Screening**
Module 5: **APPRAISE. Behind the Numbers Understanding Health Care Statistics. Science, Speed, and the Search for Best Evidence.**<br><br>*A*. Interpreting Risk<br><br>B. Misperceptions of Risk<br><br>C. Absolute and Relative Risk Reduction: How is Benefit Expressed?<br><br>D. Odds and Odds Ratios<br><br>E. Safety, Science, and Speed<br><br>F. High Dose Chemotherapy: What was the Evidence?<br><br>G. Drug Development, Early Access, and Medical Ethics	**Key concepts: Define innumeracy, discuss interpreting risk, examine misperceptions of risk and the framing effect, explain differences between absolute and relative risk, explore odds and odds ratio, social forces that can overtake good evidence, phases of drug development and early access, medical ethics and advocacy interface**<br>▪ Getting past the common problem of innumeracy<br>▪ A primer on statistical concepts relevant to health care research: different ways of presenting **risk, numerators and denominators** *(colorectal cancer example)*, **absolute risk (AR), relative risk (RR)** *(breast cancer example)*, **absolute risk reduction (risk difference) and relative risk reduction** *(Zocor example)*, **odds and odds ratios (OR)** *(hormones and memory example)*.<br>▪ Common misperceptions of risk and incidence; absolute risk and benefit.<br>▪ The **framing effect** and how risks and benefits can be exaggerated or minimized<br>▪ Health care interventions are shaped by many pressures aside from evidence<br>▪ Process of drug development is expensive, time-consuming and risky. **Phase I-IV clinical trials**<br>▪ Pressure for early access to experimental treatments can undermine research and lead to wide use of ineffective and/or harmful interventions<br>▪ Advocacy and ethical issues	***Cautionary tales:***<br><br>How public pressure and poor medical judgment can drive premature adoption of unproven treatments<br><br>▪ **High-Dose Chemotherapy with Stem Cell or Bone Marrow Transplant in Breast Cancer**<br><br>How statistics can be used for drug promotion<br><br>▪ **Zocor, tamoxifen**
Module 6: **APPLY. Making Better Decisions for Evidence Based Healthcare**<br><br>A. Elements of a Research Article<br><br>B. Critical Appraisal: Observational Study. How Do We Assess Causality	**Elements of critical appraisal, reading and interpreting a research article, associations and causality**<br>▪ What is **critical appraisal**?<br>▪ Elements of a good health care research article: **Abstract, background** and context of research, **methods**, **results**, **discussion** and context of results, **references,** and **acknowledgements**, including **conflict of interest** disclosures<br>▪ Abstracts: How to use abstracts, **structured abstracts**, uniform standards	***How to read a research article (two articles which can be downloaded):***<br><br>▪ **"A Controlled Trial of Arthroscopic Surgery for Osteoarthritis of the Knee,"** NEJM, July, 2002.<br><br>▪ **"Cigarette Smoking, Alcohol Drinking, Hepatitis B and Risk for Hepatocellular Carcinoma in**

Module	Key Concepts	Real-World Examples
C. Critical Appraisal: RCT of Arthroscopic Surgery of the Knee: Abstract, Background, Methods<br><br>D. Critical Appraisal: RCT of Arthroscopic Surgery of the Knee: Results and Discussion	▪ Methods: Ethical issues (eg, **IRB**, **informed consent**, **data monitoring**); PICO; **sample size**; randomization, allocation concealment, masking, data analysis<br>▪ Results: Participant characteristics (numbers, percentages, means and medians); Measures of association (AR, RD, RR, OR); **confidence intervals** (explained), **p-values** (explained), minimizing the effects of bias<br>▪ Discussion: Context of findings, limitations<br>▪ Acknowledgements: Industry funding issues<br>▪ Example: Liver cancer etiology (prospective cohort study). Step-by-step examination of each section of article<br>- Critical appraisal: Does abstract match full text?<br>- Associations and **causality**:<br>- Example: ecological study of colon cancer and meat consumption<br>- Are the results real or spurious? Bias, chance, confounding<br>- **Bradford-Hill criteria**: Strength (smoking and lung cancer), consistency (HIV exposure, tampons and toxic shock), specificity (smoking and many outcomes), temporality (asbestos and lung cancer), dose-response (smoking), plausibility (HIV/AIDS), experimental evidence (L-tryptophan and eosinophila-myalgia syndrome), analogy (thalidomide and congenital malformations)<br><br>▪ Example: Knee arthroscopy vs sham (RCT).<br>- Online resources for looking up medical terms<br>- Critical appraisal: Research question, ethical issues, PICO, sample size, was everybody counted **(intention to treat analysis)**?<br>- Tabular and graphical presentation of findings<br>- **Internal validity** of findings (bias, confounding), precision of estimates (95% CIs), CONSORT flow chart and standards, **generalizability** (**external validity**)<br>▪ Brief summary of course modules and wrap-up	**Korea,"** Journal of the National Cancer Institute, December, 2004
