# Supplementary material for: Development, implementation and evaluation of an online course on evidence-based healthcare for consumers
Source: BMC Health Serv Res. 2020 Oct 8;20:928. doi: 10.1186/s12913-020-05759-5 (PMC7542874; doi:10.1186/s12913-020-05759-5)
Supplement: Supplementary file 2 — Additional file 2. Survey: Participant Information – before you begin course. Survey form provided to course participants prior to accessing the course. [file 12913_2020_5759_MOESM2_ESM.pdf]

Go to Faculty Tools

Survey : Participant Information -- before you begin course

Instructions:

Thank You for your interest in the distance-education course, *Understanding Evidence-based Healthcare: A Foundation for Action,* brought to you by the United States Cochrane Center (USCC), Consumers United for Evidence-based Healthcare (CUE) and the Johns Hopkins Bloomberg School of Public Health (JHSPH). The information you provide will be used to help the USCC and CUE reach a diverse group of consumer advocates. All information is confidential.

1. What is your age? (Select one)

- Under 20
- 20 - 29
- 30 - 39
- 40 - 49
- 50 - 59
- 60 -69
- 70-79

2. How would you describe your race/ethnic origin (Select one)

Select Choice: None

○ Other: [ ]

3. What is your sex/gender? (Select one)

○ Male
○ Female
○ Prefer not to answer

4. What is the highest level of education you have completed? (Select one)

Select Choice: High school diploma or G.E.D.

5. If you selected "Professional degree" or "Doctorate" in the previous question (question #4), please specify here.

6. Are you currently... (Select one)

Select Choice: Working as a volunteer, full-time

Other:

7. Which of the following reasons best describes why you are taking this course? (Select one)

- Training (e.g. your employer has asked you to complete this course so you can use the results in training or professional development)
- Education (e.g. your educational institution has asked you to complete this course as part of a class or course of study)
- Personal growth (e.g. you have chosen to take this course for your own growth and development)
- None of the above
- Other:

8. In terms of your knowledge of evidence-based healthcare, what is your confidence level? (Select one)

- Not so confident
- Moderately confident
- Very confident

9. What is your main health area or population of focus? (Select one)

Select Choice: None

Other:

10. If you selected "Cancer" in the previous question (question #9), please specify here.

11. If you selected "Disabilities" in question #9, please specify here.

12. Are you a patient/consumer or health advocate (either paid or volunteer)? (Select one)

- ○ Yes, I work with one organization (Specify Name of Organization in question#13 and Title in question#14)
- ○ Yes, I am an independent advocate, and work with more than one organization
- ○ No

13. If you answered 'Yes, I work with one organization' to question#12, specify Name of the Organization

14. If you answered 'Yes, I work with one organization' to question#12, specify Title

15. Have you or your organization worked on issues related to evidence-based healthcare? (Select one)

- Yes (describe in question#16)
- No
- Don't know

16. If you answered 'Yes' to question#15, give a brief description

17. How confident do you feel about explaining the following concepts or defining the following terms to a friend or fellow consumer advocate (select your level of confidence where 1=low and 5=high)?

	1	2	3	4	5
Systematic review					
Evidence-based healthcare					
The Cochrane Collaboration					

How to find research articles using PubMed (MEDLINE)	○	○	○	○	○
How to use online sources (eg. The Cochrane Library) to find summaries of existing research evidence	○	○	○	○	○
Reasons why high quality systematic reviews are more useful than individual studies for understanding whether a treatment works	○	○	○	○	○
How researchers assess whether a research study's results might be due to chance	○	○	○	○	○
How to assess whether a research study's results might be explained by bias	○	○	○	○	○
Why randomizing patients in a clinical trial makes us more confident that the groups being compared are similar	○	○	○	○	○
How to assess whether an exposure might be causing an outcome or whether it might be associated with the outcome	○	○	○	○	○
Reasons why it's important that scientists publish results from ALL, not just some, of their research	○	○	○	○	○

Submit

© 2019 The Johns Hopkins University. All rights reserved.
Copyright to this collective work of materials is owned by The Johns Hopkins University.
Copyright to individual contributions may be retained by contributing authors.
