# Supplementary material for: Development, implementation and evaluation of an online course on evidence-based healthcare for consumers
Source: BMC Health Serv Res. 2020 Oct 8;20:928. doi: 10.1186/s12913-020-05759-5 (PMC7542874; doi:10.1186/s12913-020-05759-5)
Supplement: Supplementary file 3 — Additional file 3. Survey: Participant Information – after you complete course. Survey form provided to course participants after completing the course. [file 12913_2020_5759_MOESM3_ESM.pdf]

Go to Faculty Tools

Return to All Surveys

Survey : Participant Information -- after you complete course

Instructions:

Thank you for completing *Understanding Evidence-based Healthcare: A Foundation for Action*. Please respond to the brief final course assessment below. All information is confidential.

1. Name one thing that you believe you will remember from this course:

2. In terms of your current knowledge of evidence-based healthcare, now that you have completed the course, what is your confidence level?

- ◯ Not so confident
- ◯ Moderately confident
- ◯ Very confident

3. How confident do you feel about explaining the following concepts or defining the following terms to a friend or fellow consumer advocate (Select your level of confidence where 1=low and 5=high)?

	1	2	3	4	5
Systematic review	○	○	○	○	○
Evidence-based healthcare	○	○	○	○	○
The Cochrane Collaboration	○	○	○	○	○
How to find research articles using PubMed (MEDLINE)	○	○	○	○	○
How to use online sources (eg, The Cochrane Library) to find summaries of existing research evidence	○	○	○	○	○
Reasons why high quality systematic reviews are more useful than individual studies for understanding whether a treatment works	○	○	○	○	○
How researchers assess whether a research study's results might be due to chance	○	○	○	○	○
How to assess whether a research study's results might be explained by bias	○	○	○	○	○
Why randomizing patients in a clinical trial makes us more confident that the groups being compared are similar	○	○	○	○	○
How to assess whether an exposure might be causing an outcome or whether it might be associated with the outcome	○	○	○	○	○
Why it's important that scientists publish results from ALL, not just some, of their research	○	○	○	○	○

Please note that only students may submit responses. **Any responses you may submit as faculty/TA/staff do not register in the system**.

Submit

© 2019 The Johns Hopkins University. All rights reserved.
Copyright to this collective work of materials is owned by The Johns Hopkins University.
Copyright to individual contributions may be retained by contributing authors.
