# Supplementary material for: Development, implementation and evaluation of an online course on evidence-based healthcare for consumers
Source: BMC Health Serv Res. 2020 Oct 8;20:928. doi: 10.1186/s12913-020-05759-5 (PMC7542874; doi:10.1186/s12913-020-05759-5)
Supplement: Supplementary file 4 — Additional file 4. Characteristics of “Before you begin” survey completers (an expansion of published Table 1). All collected data on participant characteristics. [file 12913_2020_5759_MOESM4_ESM.pdf]

	Total completed "Before" survey		Did complete "After" survey		Did not complete "After" survey	
	No. (%)[a]		No. (%)		No. (%)	
**Age**						
Total respondents[b,c]	11 520	(100)	4 899	(100)	6 621	(100)
Under 20	150	(1.30)	36	(0.7)	114	(1.7)
20-29	3 869	(33.6)	1 788	(36.5)	2 081	(31.4)
30-39	3 059	(26.6)	1 308	(26.7)	1 751	(26.5)
40-49	2 163	(18.8)	927	(18.9)	1 236	(18.7)
50-59	1 583	(13.7)	601	(12.3)	982	(14.8)
≥60	571	(5.0)	190	(3.9)	381	(5.8)
Prefer not to answer	110	(1.0)	44	(0.9)	66	(1.0)
Not reported	15	(0.1)	5	(0.1)	10	(0.2)
**Sex/Gender**						
Total respondents	11 514	(100)	4 899	(100)	6 615	(100)
Female	8 672	(75.3)	3 927	(80.2)	4 745	(71.7)
Male	2 563	(22.3)	870	(17.8)	1 693	(25.6)
Prefer not to answer	151	(1.3)	55	(1.1)	96	(1.5)
Not reported	127	(1.1)	47	(1.0)	80	(1.2)
**Race/ethnic origin**						
Total respondents	11 522	(100)	4 899	(100)	6 623	(100)
African American/Black	1 007	(8.7)	454	(9.3)	553	(8.4)
American Indian or Alaskan Native	35	(0.3)	14	(0.3)	21	(0.3)
Asian or Pacific Islander	805	(7.0)	261	(5.3)	544	(8.2)
Caucasian/White	6 706	(58.2)	2 806	(57.3)	3 900	(58.9)
Indian/Pakistani	477	(4.1)	116	(2.4)	361	(5.5)
Latino, Latina/Hispanic	1 361	(11.8)	891	(18.2)	470	(7.1)
Middle Easterner	366	(3.2)	78	(1.6)	288	(4.4)
Other	196	(1.7)	60	(1.2)	136	(2.1)
Prefer not to answer	400	(3.5)	165	(3.4)	235	(3.6)
Not reported	169	(1.5)	54	(1.1)	115	(1.7)
**Country of residence - WHO regional classification**						
Total respondents	10 392	(100)	4 897	(100)	5 495	(100)
East Asia & Pacific	506	(4.9)	91	(1.9)	415	(7.6)
Europe & Central Asia	617	(6.0)	101	(2.1)	516	(9.4)
Latin America & Caribbean	267	(2.6)	58	(1.2)	209	(3.8)
Middle East & North Africa	285	(2.7)	44	(0.9)	241	(4.4)
North America	8 216	(79.1)	4 493	(92.8)	3 723	(67.8)
South Asia	241	(2.3)	54	(1.1)	187	(3.4)
Sub-Saharan Africa	260	(2.5)	56	(1.1)	204	(3.7)
**Country of residence - WHO economy classification**						
Total respondents	10 392	(100)	4 897	(100)	5 495	(100)
High income	9 319	(89.7)	4 685	(95.7)	4 634	(84.3)
Upper middle income	356	(3.4)	75	(1.5)	281	(5.1)
Lower middle income	628	(6.0)	120	(2.5)	508	(9.2)
Low income	89	(0.9)	17	(0.4)	72	(1.3)

	Total completed "Before" survey		Did complete "After" survey		Did not complete "After" survey	
	No. (%)[a]		No. (%)		No. (%)	
Highest level of education completed no. (%)						
Total respondents	11 510	(100)	4 899	(100)	6 611	(100)
Some high school	56	(0.5)	17	(0.4)	39	(0.6)
High school diploma or G.E.D.	294	(2.6)	102	(2.1)	192	(2.9)
Some college (no degree)	982	(8.5)	473	(9.7)	509	(7.7)
Trade/technical school	77	(0.7)	27	(0.6)	50	(0.8)
Associate degree	1 209	(10.5)	778	(15.9)	431	(6.5)
Bachelor's degree	4 288	(37.3)	2 038	(41.6)	2 250	(34.0)
Master's degree	2 133	(18.5)	650	(13.3)	1 483	(22.4)
Professional degree (e.g., DDS, JD, RN, BSN)	1 517	(13.2)	583	(11.9)	934	(14.1)
Doctorate (e.g., PhD, EdD)	749	(6.5)	163	(3.3)	586	(8.9)
Prefer not to answer	158	(1.4)	49	(1.0)	109	(1.7)
No response	47	(0.4)	19	(0.4)	28	(0.4)
Employment status, no. (%)						
Total respondents	11 522	(100)	4 899	(100)	6 623	(100)
Employed full-time for an income	5 651	(49.1)	2 479	(50.6)	3 172	(47.9)
Employed part-time for an income	1 726	(15.0)	785	(16.0)	941	(14.2)
Working as a volunteer, full-time	47	(0.4)	10	(0.2)	37	(0.6)
Working as a volunteer, part-time	113	(1.0)	46	(1.0)	67	(1.0)
Employed for an income and working as a volunteer	294	(2.6)	102	(2.1)	192	(2.9)
Not employed or not working, student	1 916	(16.6)	796	(16.3)	1 120	(16.9)
Not employed or not working, other	86	(1.3)	37	(0.8)	123	(1.1)
Other	1 214	(10.5)	490	(10.0)	724	(10.9)
Prefer not to answer	274	(2.4)	85	(1.7)	189	(2.9)
Not reported	164	(1.4)	69	(1.4)	95	(1.4)
Reason for taking the course, no. (%)						
Total respondents	11 522	(100)	4 899	(100)	6 623	(100)
Training	846	(7.3)	272	(5.6)	574	(8.7)
Education	6 412	(55.7)	3 782	(77.2)	2 930	(39.7)
Personal growth	3 451	(30.0)	584	(11.9)	2 867	(43.3)
None of the above	113	(1.0)	17	(0.4)	96	(1.5)
Other	566	(4.9)	180	(3.7)	386	(5.8)
Not reported	134	(1.2)	64	(1.3)	70	(1.1)

	Total completed "Before" survey No. (%)[a]		Did complete "After" survey No. (%)		Did not complete "After" survey No. (%)	
Main health area or population of focus, no. (%)						
Aging	362	(3.1)	173	(3.5)	189	(2.9)
Blood, heart and circulation	356	(3.1)	164	(3.4)	192	(2.9)
Bones, joints and muscles	350	(3.0)	124	(2.5)	226	(3.4)
Cancer	899	(7.8)	398	(8.1)	501	(7.6)
Child and teen health	540	(4.7)	243	(5.0)	297	(4.5)
Critical care and intensive care	517	(4.5)	303	(6.2)	214	(3.2)
Diabetes	186	(1.6)	79	(1.6)	107	(1.6)
Family medicine and primary care	574	(5.0)	254	(5.2)	320	(4.8)
General consumer issues	131	(2.0)	65	(1.3)	196	(1.7)
HIV/AIDS	127	(1.1)	35	(0.7)	92	(1.4)
Health disparities and minority health	120	(1.0)	32	(0.7)	88	(1.3)
Mental health	337	(2.9)	141	(2.9)	196	(3.0)
Nursing	1 918	(16.7)	1 173	(23.9)	745	(11.3)
Pregnancy and reproduction	453	(3.9)	126	(2.6)	327	(4.9)
Public health	552	(4.8)	122	(2.5)	430	(6.5)
Women's health	526	(4.6)	226	(4.6)	300	(4.5)
Other[d]	3 120	(27.1)	1 098	(22.4)	2 022	(30.5)
Not reported	389	(3.4)	143	(2.9)	246	(3.7)
Patient/consumer or health advocate (either paid or volunteer)						
Yes, I work with one organization	1 902	(16.5)	825	(16.8)	1 077	(16.4)
Yes, I am an independent advocate, and work with more than one organization	776	(6.8)	253	(5.2)	523	(7.9)
No	8 434	(73.4)	3 672	(75.0)	4 762	(72.2)
Not reported	384	(3.3)	149	(3.0)	235	(3.6)
Experience working on issues related to evidence-based healthcare						
Yes	2 988	(26.0)	1 095	(22.4)	1 893	(28.8)
No	4 558	(39.7)	1 998	(40.8)	2 560	(38.9)
Don't know	3 283	(28.6)	1 559	(31.8)	1 724	(26.2)
No response given	653	(5.7)	247	(5.0)	406	(6.2)

[a] Columns may not total 100% owing to rounding

[b] Although the online hosting platform prompted participants to answer all survey questions before proceeding, we still encountered missing responses

[c] All items are self-reported

[d] Participants submitted over 1 100 unique health areas under "Other", 98% of which had 10 counts or fewer.
